# Dichloridobis(1,10-phenanthroline-5,6-dione-κ^2^ *N*,*N*′)mercury(II)

**DOI:** 10.1107/S1600536809049289

**Published:** 2009-11-21

**Authors:** Carlos A. L. Figueiras, João A. S. Bomfim, R. Alan Howie, Edward R. T. Tiekink, James L. Wardell

**Affiliations:** a Departamento de Química Inorgânica, Instituto de Química, Universidade Federal do Rio de Janeiro, CP 68563, 21941-909 Rio de Janeiro, RJ, Brazil; b Department of Chemistry, University of Aberdeen, Old Aberdeen, AB15 5NY, Scotland; c Department of Chemistry, University of Malaya, 50603 Kuala Lumpur, Malaysia; d Departamento de Quimica, ICEx, Universidade Federal de Minas Gerais, 31270-901 Belo Horizonte, MG, Brazil

## Abstract

In the title compound, [HgCl_2_(C_12_H_6_N_2_O_2_)_2_], the Hg^II^ atom is located on a twofold rotation axis and exists within a distorted octa­hedral geometry defined by a *cis*-Cl_2_N_4_ donor set. Mol­ecules are connected into layers in the *ac* plane *via* extensive C—H⋯Cl contacts as each Cl atom forms two such inter­actions. Contacts between the layers are of the type C=O⋯π [O⋯centroid distance = 3.110 (8) Å].

## Related literature

For related main-group compounds of 1,10-phenanthroline-5,6-dione, see: de Alencastro *et al.* (2005 ▶). For the ligand synthesis, see: Yamada *et al.* (1992 ▶). For a related structure, see: Ramezanipour *et al.* (2005 ▶).
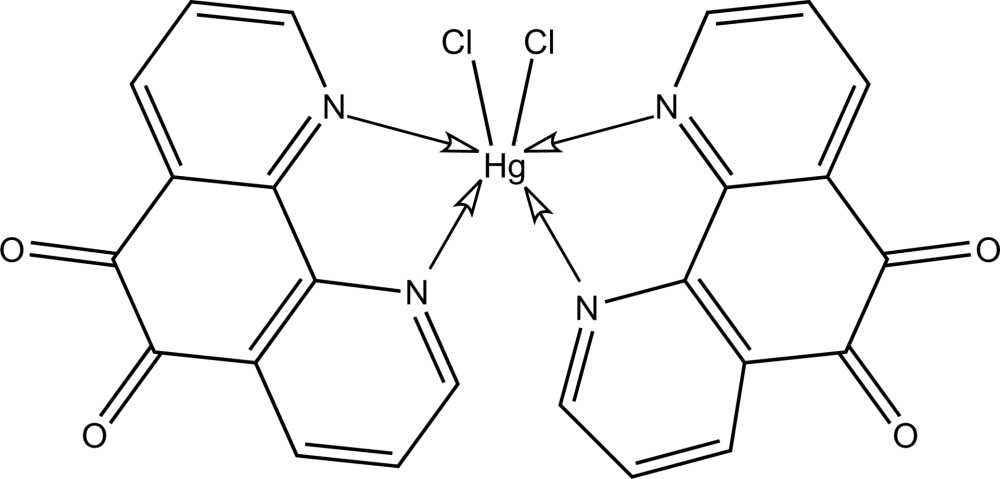


## Experimental

### 

#### Crystal data

[HgCl_2_(C_12_H_6_N_2_O_2_)_2_]
*M* *_r_* = 691.87
Orthorhombic, 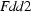

*a* = 8.2261 (2) Å
*b* = 42.6761 (11) Å
*c* = 12.6108 (3) Å
*V* = 4427.12 (19) Å^3^
*Z* = 8
Mo *K*α radiation
μ = 7.24 mm^−1^
*T* = 120 K
0.20 × 0.10 × 0.06 mm

#### Data collection

Nonius KappaCCD diffractometer
Absorption correction: multi-scan (*SADABS*; Sheldrick, 1996 ▶) *T*_min_ = 0.204, *T*_max_ = 0.651
10751 measured reflections
2487 independent reflections
2239 reflections with *I* > 2σ(*I*)
*R*_int_ = 0.075

#### Refinement

*R*[*F* ^2^ > 2σ(*F* ^2^)] = 0.037
*wR*(*F* ^2^) = 0.094
*S* = 1.05
2487 reflections
159 parameters
1 restraint
H-atom parameters constrained
Δρ_max_ = 2.27 e Å^−3^
Δρ_min_ = −0.68 e Å^−3^
Absolute structure: Flack (1983 ▶), 1163 Friedel pairs
Flack parameter: −0.012 (13)

### 

Data collection: *COLLECT* (Hooft, 1998 ▶); cell refinement: *DENZO* (Otwinowski & Minor, 1997 ▶) and *COLLECT*; data reduction: *DENZO* and *COLLECT*; program(s) used to solve structure: *SHELXS97* (Sheldrick, 2008 ▶); program(s) used to refine structure: *SHELXL97* (Sheldrick, 2008 ▶); molecular graphics: *DIAMOND* (Brandenburg, 1999 ▶); software used to prepare material for publication: *publCIF* (Westrip, 2009 ▶).

## Supplementary Material

Crystal structure: contains datablocks global, I. DOI: 10.1107/S1600536809049289/hy2254sup1.cif

Structure factors: contains datablocks I. DOI: 10.1107/S1600536809049289/hy2254Isup2.hkl

Additional supplementary materials:  crystallographic information; 3D view; checkCIF report

## Figures and Tables

**Table 1:** Selected bond lengths (Å)

Hg—N1	2.439 (7)
Hg—N2	2.512 (6)
Hg—Cl1	2.5270 (19)

**Table 2:** Hydrogen-bond geometry (Å, °)

*D*—H⋯*A*	*D*—H	H⋯*A*	*D*⋯*A*	*D*—H⋯*A*
C2—H2⋯Cl1^i^	0.95	2.69	3.578 (8)	156
C9—H9⋯Cl1^ii^	0.95	2.78	3.701 (8)	164

Symmetry codes: (i) 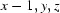; (ii) 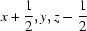.

**Fig. 1.**
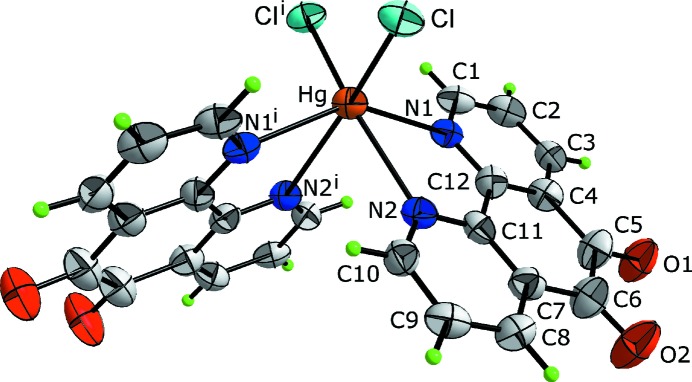
Molecular structure of the title compound. Displacement ellipsoids are drawn at the 50% probability level. [Symmetry code: (i) -x, -y, z.]

**Fig. 2.**
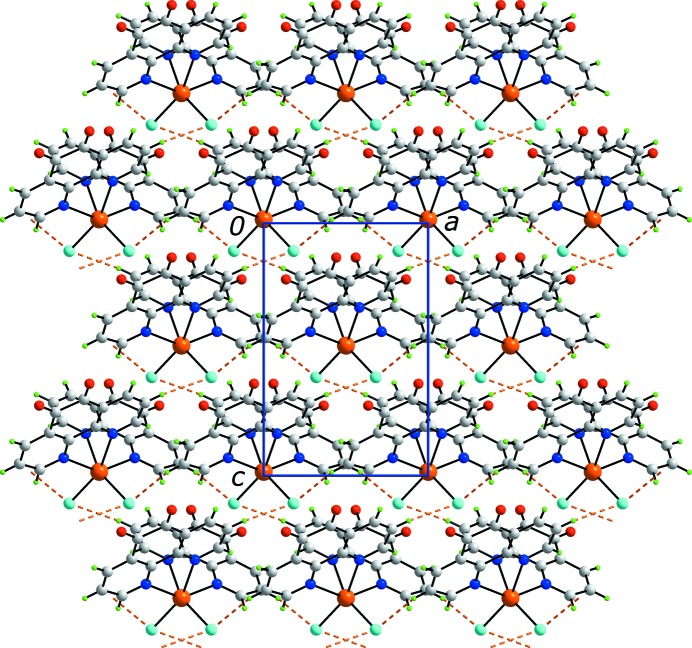
Supramolecular array in the ac plane mediated by C—H···Cl contacts (orange dashed lines). [Colour codes: Hg orange; Cl cyan; O red; N blue; C grey; H green.]

**Fig. 3.**
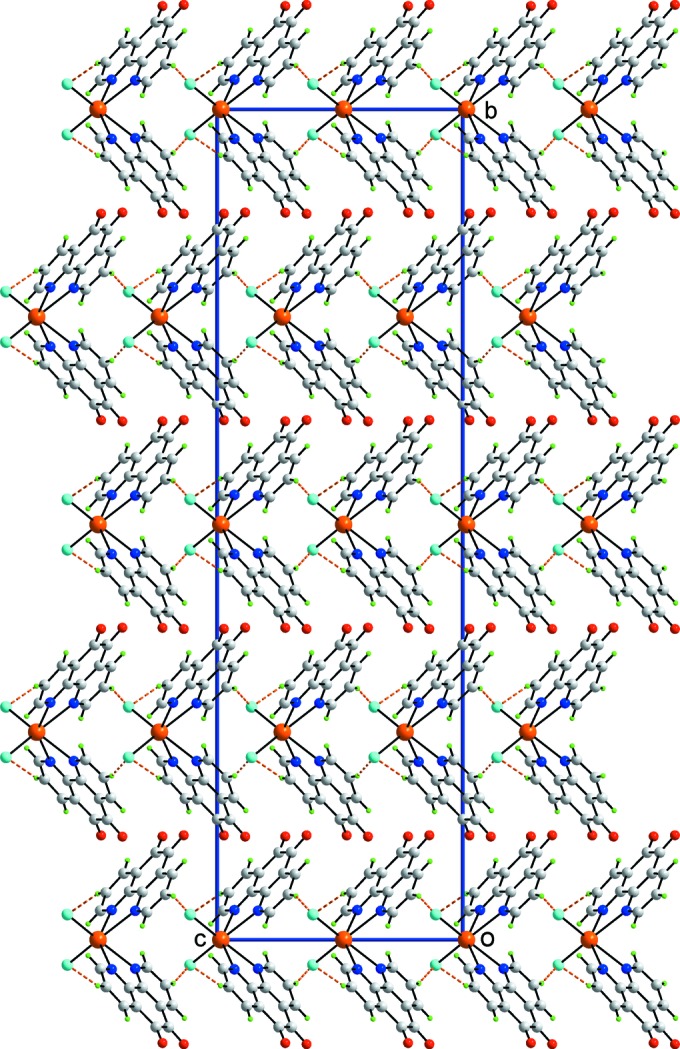
Unit-cell contents viewed in projection down the a axis. The C—H···Cl contacts are shown as orange dashed lines. [Colour codes: Hg orange; Cl cyan; O red; N blue; C grey; H green.]

## supplementary crystallographic information

### Comment

In continuation of the previous studies of coordination compounds of 1,10-phenanthroline-5,6-dione (pdon) ligand (de Alencastro *et al.*, 2005), the title compound, HgCl_2_(pdon)_2_, was investigated. The reaction of HgCl_2_ with pdon (1:1 mol ratio) led to the isolation of a pure 1:1 complex, HgCl_2_(pdon). However, as the product was unsuitable for X-ray crystallography, a further recrystallization from MeNO_2_ solution was attempted, which led to the isolation of the title compound, a 1:2 complex. Of interest was that the 1:1 HgI_2_(pdon) complex, prepared similarly to the chloride analogue, was recovered on recrystallization from MeNO_2_. Unfortunately this set of crystals also proved unsuitable for X-ray crystallography.

In the title compound, the Hg^II^ atom lies on a crystallographic twofold axis and exists within a *cis*-Cl_2_N_4_ donor set defined by two Cl atoms and four N atoms derived from two chelating pdon ligands (Fig. 1). There is a small disparity in the magnitude of the Hg—N bond distances, with Hg—N1 of 2.439 (7) Å being shorter than Hg—N2 of 2.512 (6) Å. Distortions from an ideal octahedral geometry are related in part to the acute chelate angle of 66.9 (2)°. The pdon ligand is essentially planar with a RMS of 0.226 Å for the N and C atoms, with O1 and O2 atoms lying, respectively, -0.079 (12) and 0.071 (13) Å out of the least-squares plane. The dihedral angle formed between the symmetry related 1,10-phenanthroline planes is 87.13 (11)°. The structural features described herein for the title compound resemble those found for HgCl_2_(1,10-phenanthroline)_2_ (Ramezanipour *et al.*, 2005).

In the crystal structure, C—H···Cl interactions are found so that each Cl atom is associated with two H atoms to form supramolecular arrays in the *ac* plane (Table 1 and Fig. 2). The most prominent interactions between the layers are of the type C═O···π. The closest of these involves the carbonyl-O1 group and the centroid (*Cg*) of C4–C7, C11, C12 ring [O1···*Cg*^i^ = 3.110 (8) Å with the C5–O1···*Cg*^i^ angle being 133.1 (8)°, symmetry code: (i) -1/4+x, 1/4-y, -1/4+z] (Fig. 3).

### Experimental

Solutions of HgCl_2_ (0.272 mg, 1.0 mmol) in EtOH (5 ml) and of 1,10-phenanthroline-5,6-dione (Yamada *et al.*, 1992) (0.212 mg, 1.0 mmol) in EtOH (20 ml) were mixed and stirred at room temperature for 1 h. The precipitate was collected, washed with small portions of EtOH and petroleum ether and dried (yield 0.330 mg). Analysis, calculated for C_12_H_6_Cl_2_HgN_2_O_2_ [HgCl_2_(pdon)]: C 29.9, H 1.3, N 5.8%; found: C 30.2, H 1.5, N 5.2%.

Recrystallization of HgCl_2_(pdon) from MeNO_2_ solution produced crystals of the title compound, HgCl_2_(pdon)_2_.

### Refinement

H atoms were geometrically placed with C—H = 0.95 Å, and refined as riding with *U*_iso_(H) = 1.2*U*_eq_(C). The maximum and minimum residual electron density peaks of 2.27 and 0.68 e Å^-3^ were located 0.91 and 0.82 Å from Hg atom, respectively.

### Crystal data

**Table:**

[HgCl_2_(C_12_H_6_N_2_O_2_)_2_]	*F*(000) = 2640
*M**_r_* = 691.87	*D*_x_ = 2.076 Mg m^−^^3^
Orthorhombic, *F**d**d*2	Mo *K*α radiation, λ = 0.71073 Å
Hall symbol: F 2 -2d	Cell parameters from 18660 reflections
*a* = 8.2261 (2) Å	θ = 2.9–27.5°
*b* = 42.6761 (11) Å	µ = 7.24 mm^−^^1^
*c* = 12.6108 (3) Å	*T* = 120 K
*V* = 4427.12 (19) Å^3^	Block, yellow
*Z* = 8	0.20 × 0.10 × 0.06 mm

### Data collection

**Table:**

Nonius KappaCCD diffractometer	2487 independent reflections
Radiation source: Enraf Nonius FR591 rotating anode	2239 reflections with *I* > 2σ(*I*)
10 cm confocal mirrors	*R*_int_ = 0.075
Detector resolution: 9.091 pixels mm^-1^	θ_max_ = 27.5°, θ_min_ = 3.0°
φ and ω scans	*h* = −10→8
Absorption correction: multi-scan (*SADABS*; Sheldrick, 1996)	*k* = −55→55
*T*_min_ = 0.204, *T*_max_ = 0.651	*l* = −16→16
10751 measured reflections	

### Refinement

**Table:**

Refinement on *F*^2^	Secondary atom site location: difference Fourier map
Least-squares matrix: full	Hydrogen site location: inferred from neighbouring sites
*R*[*F*^2^ > 2σ(*F*^2^)] = 0.037	H-atom parameters constrained
*wR*(*F*^2^) = 0.094	*w* = 1/[σ^2^(*F*_o_^2^) + (0.0564*P*)^2^ + 3.1352*P*] where *P* = (*F*_o_^2^ + 2*F*_c_^2^)/3
*S* = 1.05	(Δ/σ)_max_ < 0.001
2487 reflections	Δρ_max_ = 2.27 e Å^−^^3^
159 parameters	Δρ_min_ = −0.68 e Å^−^^3^
1 restraint	Absolute structure: Flack (1983), 1163 Friedel pairs
Primary atom site location: structure-invariant direct methods	Flack parameter: −0.012 (13)

### Fractional atomic coordinates and isotropic or equivalent isotropic displacement parameters (Å^2^)

**Table:**

	*x*	*y*	*z*	*U*_iso_*/*U*_eq_	
Hg	0.0000	0.0000	0.48512 (8)	0.04052 (12)	
Cl1	0.1811 (2)	0.03035 (5)	0.61020 (15)	0.0486 (4)	
O1	−0.3649 (8)	0.12440 (15)	0.2228 (6)	0.072 (2)	
O2	−0.0592 (10)	0.12581 (18)	0.1361 (7)	0.081 (2)	
N1	−0.2157 (8)	0.03565 (16)	0.4299 (5)	0.0406 (14)	
N2	0.0815 (7)	0.03486 (14)	0.3337 (4)	0.0399 (13)	
C1	−0.3618 (9)	0.03505 (19)	0.4754 (6)	0.0472 (16)	
H1	−0.3840	0.0191	0.5258	0.057*	
C2	−0.4837 (9)	0.0567 (2)	0.4528 (7)	0.050 (2)	
H2	−0.5869	0.0554	0.4864	0.060*	
C3	−0.4507 (11)	0.07993 (18)	0.3806 (6)	0.0459 (17)	
H3	−0.5300	0.0955	0.3653	0.055*	
C4	−0.3014 (10)	0.08061 (18)	0.3300 (6)	0.0435 (16)	
C5	−0.2664 (10)	0.1051 (2)	0.2493 (14)	0.057 (3)	
C6	−0.0923 (11)	0.1052 (2)	0.1979 (8)	0.059 (2)	
C7	0.0217 (9)	0.0800 (2)	0.2297 (7)	0.0450 (17)	
C8	0.1742 (11)	0.0790 (2)	0.1830 (6)	0.0482 (18)	
H8	0.2071	0.0945	0.1335	0.058*	
C9	0.2777 (9)	0.0545 (2)	0.2110 (6)	0.0453 (18)	
H9	0.3818	0.0526	0.1792	0.054*	
C10	0.2257 (10)	0.03325 (19)	0.2861 (7)	0.0437 (17)	
H10	0.2965	0.0166	0.3047	0.052*	
C11	−0.0196 (9)	0.05763 (18)	0.3047 (6)	0.0409 (16)	
C12	−0.1850 (9)	0.05809 (16)	0.3578 (7)	0.0413 (14)	

### Atomic displacement parameters (Å^2^)

**Table:**

	*U*^11^	*U*^22^	*U*^33^	*U*^12^	*U*^13^	*U*^23^
Hg	0.03662 (19)	0.04817 (18)	0.03677 (16)	0.00021 (19)	0.000	0.000
Cl1	0.0400 (9)	0.0635 (11)	0.0424 (9)	−0.0042 (8)	−0.0003 (8)	−0.0077 (8)
O1	0.062 (4)	0.064 (4)	0.091 (5)	0.015 (3)	0.005 (4)	0.029 (4)
O2	0.055 (4)	0.082 (5)	0.106 (6)	0.016 (4)	0.016 (4)	0.049 (4)
N1	0.042 (3)	0.046 (3)	0.034 (3)	−0.003 (3)	0.004 (2)	−0.003 (3)
N2	0.035 (3)	0.049 (3)	0.036 (3)	0.003 (3)	0.000 (2)	−0.001 (2)
C1	0.039 (4)	0.064 (4)	0.039 (3)	−0.007 (3)	−0.002 (3)	−0.005 (4)
C2	0.033 (4)	0.069 (5)	0.047 (4)	−0.006 (3)	0.001 (3)	−0.010 (3)
C3	0.044 (4)	0.047 (4)	0.047 (4)	0.006 (3)	−0.004 (3)	−0.011 (3)
C4	0.032 (3)	0.053 (4)	0.045 (4)	0.001 (3)	−0.006 (3)	−0.004 (3)
C5	0.048 (5)	0.055 (4)	0.067 (10)	0.002 (4)	−0.011 (5)	0.012 (5)
C6	0.052 (5)	0.059 (5)	0.067 (5)	0.000 (4)	−0.008 (4)	0.012 (4)
C7	0.044 (4)	0.048 (4)	0.043 (4)	−0.003 (3)	0.004 (3)	−0.001 (3)
C8	0.050 (5)	0.052 (4)	0.043 (4)	−0.004 (3)	−0.001 (3)	0.007 (3)
C9	0.037 (4)	0.062 (5)	0.038 (4)	−0.011 (3)	0.001 (3)	−0.007 (3)
C10	0.047 (5)	0.042 (4)	0.042 (4)	0.005 (3)	−0.007 (3)	−0.001 (3)
C11	0.043 (4)	0.042 (4)	0.038 (3)	0.000 (3)	−0.002 (3)	−0.009 (3)
C12	0.039 (4)	0.040 (3)	0.045 (3)	0.000 (3)	−0.009 (3)	−0.002 (3)

### Geometric parameters (Å, °)

**Table:**

Hg—N1	2.439 (7)	C3—H3	0.9500
Hg—N2	2.512 (6)	C4—C12	1.401 (11)
Hg—Cl1	2.5270 (19)	C4—C5	1.487 (15)
O1—C5	1.203 (11)	C5—C6	1.572 (14)
O2—C6	1.206 (11)	C6—C7	1.483 (12)
N1—C1	1.332 (10)	C7—C11	1.386 (12)
N1—C12	1.344 (10)	C7—C8	1.387 (12)
N2—C11	1.330 (10)	C8—C9	1.392 (12)
N2—C10	1.331 (10)	C8—H8	0.9500
C1—C2	1.393 (12)	C9—C10	1.380 (11)
C1—H1	0.9500	C9—H9	0.9500
C2—C3	1.374 (13)	C10—H10	0.9500
C2—H2	0.9500	C11—C12	1.516 (11)
C3—C4	1.384 (11)		
			
N1—Hg—N1^i^	146.8 (3)	C4—C3—H3	120.1
N1—Hg—N2^i^	87.6 (2)	C3—C4—C12	118.4 (8)
N1^i^—Hg—N2^i^	66.9 (2)	C3—C4—C5	120.1 (7)
N1—Hg—N2	66.9 (2)	C12—C4—C5	121.4 (7)
N1^i^—Hg—N2	87.6 (2)	O1—C5—C4	122.8 (9)
N2^i^—Hg—N2	81.0 (3)	O1—C5—C6	119.8 (11)
N1—Hg—Cl1	106.71 (16)	C4—C5—C6	117.4 (8)
N1^i^—Hg—Cl1	93.95 (16)	O2—C6—C7	124.1 (9)
N2^i^—Hg—Cl1	159.31 (13)	O2—C6—C5	118.3 (9)
N2—Hg—Cl1	90.78 (14)	C7—C6—C5	117.5 (8)
N1—Hg—Cl1^i^	93.95 (16)	C11—C7—C8	119.4 (8)
N1^i^—Hg—Cl1^i^	106.71 (16)	C11—C7—C6	122.0 (7)
N2^i^—Hg—Cl1^i^	90.78 (14)	C8—C7—C6	118.6 (8)
N2—Hg—Cl1^i^	159.31 (13)	C7—C8—C9	117.9 (8)
Cl1—Hg—Cl1^i^	102.75 (10)	C7—C8—H8	121.0
C1—N1—C12	118.3 (7)	C9—C8—H8	121.0
C1—N1—Hg	121.4 (5)	C10—C9—C8	118.6 (7)
C12—N1—Hg	120.0 (5)	C10—C9—H9	120.7
C11—N2—C10	118.1 (7)	C8—C9—H9	120.7
C11—N2—Hg	118.3 (5)	N2—C10—C9	123.5 (7)
C10—N2—Hg	123.4 (5)	N2—C10—H10	118.3
N1—C1—C2	123.3 (8)	C9—C10—H10	118.3
N1—C1—H1	118.4	N2—C11—C7	122.5 (7)
C2—C1—H1	118.4	N2—C11—C12	116.7 (7)
C3—C2—C1	118.2 (7)	C7—C11—C12	120.8 (7)
C3—C2—H2	120.9	N1—C12—C4	122.0 (8)
C1—C2—H2	120.9	N1—C12—C11	117.3 (7)
C2—C3—C4	119.8 (7)	C4—C12—C11	120.7 (7)
C2—C3—H3	120.1		
			
N1^i^—Hg—N1—C1	135.2 (6)	C4—C5—C6—C7	2.9 (16)
N2^i^—Hg—N1—C1	96.6 (6)	O2—C6—C7—C11	177.0 (10)
N2—Hg—N1—C1	177.8 (6)	C5—C6—C7—C11	−2.3 (14)
Cl1—Hg—N1—C1	−98.6 (6)	O2—C6—C7—C8	−2.6 (15)
Cl1^i^—Hg—N1—C1	5.9 (6)	C5—C6—C7—C8	178.0 (9)
N1^i^—Hg—N1—C12	−50.0 (5)	C11—C7—C8—C9	2.5 (12)
N2^i^—Hg—N1—C12	−88.6 (6)	C6—C7—C8—C9	−177.8 (8)
N2—Hg—N1—C12	−7.4 (5)	C7—C8—C9—C10	−2.2 (12)
Cl1—Hg—N1—C12	76.2 (6)	C11—N2—C10—C9	2.3 (12)
Cl1^i^—Hg—N1—C12	−179.2 (5)	Hg—N2—C10—C9	−172.5 (6)
N1—Hg—N2—C11	7.4 (5)	C8—C9—C10—N2	−0.2 (12)
N1^i^—Hg—N2—C11	165.7 (5)	C10—N2—C11—C7	−1.9 (11)
N2^i^—Hg—N2—C11	98.7 (5)	Hg—N2—C11—C7	173.2 (6)
Cl1—Hg—N2—C11	−100.4 (5)	C10—N2—C11—C12	177.9 (7)
Cl1^i^—Hg—N2—C11	30.9 (8)	Hg—N2—C11—C12	−7.0 (8)
N1—Hg—N2—C10	−177.7 (6)	C8—C7—C11—N2	−0.5 (12)
N1^i^—Hg—N2—C10	−19.5 (6)	C6—C7—C11—N2	179.8 (8)
N2^i^—Hg—N2—C10	−86.5 (6)	C8—C7—C11—C12	179.7 (7)
Cl1—Hg—N2—C10	74.4 (6)	C6—C7—C11—C12	0.0 (12)
Cl1^i^—Hg—N2—C10	−154.2 (5)	C1—N1—C12—C4	0.1 (11)
C12—N1—C1—C2	−0.6 (12)	Hg—N1—C12—C4	−174.9 (5)
Hg—N1—C1—C2	174.3 (6)	C1—N1—C12—C11	−178.1 (6)
N1—C1—C2—C3	−0.5 (12)	Hg—N1—C12—C11	6.9 (9)
C1—C2—C3—C4	2.2 (12)	C3—C4—C12—N1	1.6 (11)
C2—C3—C4—C12	−2.7 (11)	C5—C4—C12—N1	−179.4 (9)
C2—C3—C4—C5	178.2 (9)	C3—C4—C12—C11	179.8 (7)
C3—C4—C5—O1	−2.1 (18)	C5—C4—C12—C11	−1.2 (13)
C12—C4—C5—O1	178.8 (11)	N2—C11—C12—N1	0.3 (10)
C3—C4—C5—C6	177.9 (9)	C7—C11—C12—N1	−179.9 (7)
C12—C4—C5—C6	−1.1 (16)	N2—C11—C12—C4	−178.0 (7)
O1—C5—C6—O2	3.5 (19)	C7—C11—C12—C4	1.9 (11)
C4—C5—C6—O2	−176.5 (10)	O1—C5—C6—O2	3.5 (19)
O1—C5—C6—C7	−177.1 (11)		

Symmetry codes: (i) −*x*, −*y*, *z*.

### Hydrogen-bond geometry (Å, °)

**Table:**

*D*—H···*A*	*D*—H	H···*A*	*D*···*A*	*D*—H···*A*
C2—H2···Cl1^ii^	0.95	2.69	3.578 (8)	156
C9—H9···Cl1^iii^	0.95	2.78	3.701 (8)	164

Symmetry codes: (ii) *x*−1, *y*, *z*; (iii) *x*+1/2, *y*, *z*−1/2.
